# Why Are We Weary?

**Published:** 1894-10

**Authors:** 


### SELECTIONS.

### Why are We Weary?

Work and want of work, bring weariness. Hot haste and hot rooms bring fatigue. There are moral causes and there are physical—the moral produces the physical. But why do these do so? Dr. Hodge, of Clark University, has been for some years making experiments with a view of elucidating the pathology, or explaining the physiology, of fatigue. He refers to the fact that all the energy of the body comes directly from chemical changes and reactions in the individual cells of which the body is composed. If we could know the processes which take place in a single nerve cell, it would be the key to the whole of nerve physiology, from the action of the nervous mechanism in the protoplasm of the amœba, and thence through the entire animal series, to the activity of the human brain. It has been well known that every kind of cell has its peculiar function—gland cells to secrete their own fluid, the cells of muscle to produce work, whereas nerve cells produce what we call consciousness—a thing outside of physical observation.

But the nerve cells have the same life-history of birth, maturity, and death, as other cells; hence they suffer from want of nourishment, fatigue, and in the struggle for existence.

Dr. Hodge has been trying to find out what changes occurred in the cells of healthy animals, by passing electric currents through them. Various animals were used for this purpose, and after they were killed the nerve cells were examined under the microscope.

Some of the animals were put under the influence of ether, and a mild current carried directly to the one side of the optic thalamus and other sections of the brain, and after a time the animal was killed and opposite sections made and examined. The results of a large number of experiments exhibited a uniform diminution and decrease of the size of the nucleus of the cells, which had been subjected to the stimulation, and a change from smooth and round to jagged, irregular outlines. The recticular appearance was broken up, the cell protoplasm was shrunken in size, and the nuclei were decreased in size. The sections of brain not stimulated showed no changes of this nature. Great care was taken that the electrical current should be within physiologic limits. This inquiry followed: Do similar changes occur in the normal activity of an animal from fatigue? and are they restored by rest and sleep? To answer this numerous sparrows, pigeons and swallows were shot in the morning, as they were going out, and at night when coming home after a day of activity. Sections of similar parts of the brains of the morning and evening birds were compared with each other. In all cases the brain cells of the birds at evening were greatly decreased in size, and the nucleus jagged and of irregular outline. The cell protoplasm was also shrunken and reticulated in appearance. The nuclei were also greatly decreased. Honey bees were caught coming out of the hive in the morning and compared with the bees going in late at night. The same differences were noted in the brain cells, showing degrees of exhaustion and fatigue in one, and vigor and health in the others.

These studies, conducted with great accuracy and detail for the purpose of eliminating all errors, seem to point to brain and nerve fatigue as a shrinking of the nucleus and cell protoplasm. Birds indentical in appearance, shot at night and in the morning, brought out this fact, in the shrunken cells at night, and the full rounded cells in the morning. The conclusion is that in sleep the wasted cell recovers its functional changes from fatigue, and the failure of this restoration is the beginning of organic degeneration of all forms and degrees. The cell recovers the bundles of nerve fibers which enter into it, and are lost in the granular substance and nucleus. Here all the impressions of the external world come in a succession of impulses, and all the outgoing commands are determined.

Something like this decreased and shrinking of cell contents and nucleus are common in old age. It would appear reasonable to suppose that failure to restore the cell contents would be ageing and practical starvation of the body. If physiological fatigue is functional changes in nerve cells which can be restored by rest and sleep, scientific inquiry has taken a step forward of deep significance.

Dr. Hodge has been for some time making studies of the changes which take place in nerve cells under variations of food and water supply. Also what changes if any take place in nerve cells from birth to death from old age, from rejuvenation to senescence. These researches will be watched with much interest.—*The Popular Medical Monthly.*

